# Prospective evaluation of accuracy and clinical utility of the Dual Path Platform (DPP) assay for the point-of-care diagnosis of leptospirosis in hospitalized patients

**DOI:** 10.1371/journal.pntd.0006285

**Published:** 2018-02-20

**Authors:** Scott A. Nabity, José E. Hagan, Guilherme Araújo, Alcinéia O. Damião, Jaqueline S. Cruz, Nivison Nery, Elsio A. Wunder, Mitermayer G. Reis, Albert I. Ko, Guilherme S. Ribeiro

**Affiliations:** 1 Departments of Medicine and Pediatrics, Massachusetts General Hospital, Harvard Medical School, Boston, United States of America; 2 Laboratório de Patologia e Biologia Molecular, Instituto Gonçalo Moniz, Fundação Oswaldo Cruz, Salvador, Brazil; 3 Department of Epidemiology of Microbial Diseases, Yale School of Public Health, New Haven, United States of America; 4 Departamento de Saúde Coletiva, Instituto de Saúde Coletiva, Universidade Federal da Bahia, Salvador, Brazil; Medical College of Wisconsin, UNITED STATES

## Abstract

Early detection of leptospirosis with field-ready diagnostics may improve clinical management and mitigate outbreaks. We previously validated the point-of-care Dual Path Platform (DPP) for leptospirosis with sera in the laboratory. This prospective study compares the diagnostic accuracy and clinical utility of the DPP using finger stick blood (FSB) against the serum DPP, venous whole blood (VWB) DPP, IgM-ELISA, and clinical impression. We sequentially enrolled 98 patients hospitalized for acute febrile illnesses, of which we confirmed 32 by leptospirosis reference tests. Among syndromes consistent with classic leptospirosis, the FSB DPP showed similar sensitivity and specificity (Se 93% and Sp 80%), and positive and negative predictive values (PPV 74% and NPV 95%), to VWB DPP (Se 96%, Sp 75%, PPV 68%, and NPV 97%), serum DPP (Se 85%, Sp 87%, PPV 79%, and NPV 91%) and IgM-ELISA (Se 81%, Sp 100%, PPV 100%, and NPV 90%). The FSB DPP provided a favorable likelihood ratio profile (positive LR 4.73, negative LR 0.09) in comparison to other assays and clinical impression alone. Additionally, we identified four of five leptospirosis-associated meningitis patients by whole blood DPP, none of which clinicians suspected. This demonstrates potential for the DPP in routine detection of this less common syndrome. The FSB DPP demonstrated similar discrimination for severe human leptospirosis compared with serum assays, and it is a simpler option for diagnosing leptospirosis. Its performance in other epidemiological settings and geographic regions, and for detecting atypical presentations, demands further evaluation.

## Author summary

The reliable, portable, point-of-care DPP assay effectively discriminates case status for patients presenting to hospital with acute febrile syndromes consistent with classic leptospirosis. Diagnostic accuracy of the finger stick DPP using the initial acute-phase specimen at the bedside is similar to serum DPP and IgM-ELISA, yet diagnosticians can perform the DDP assay in 20 minutes without laboratory equipment. The finger stick DPP expands rapid diagnostic options at the bedside for severe leptospirosis in humans.

## Introduction

Leptospirosis is an important global cause of acute fever and a leading cause of morbidity among zoonotic diseases [1]. Annually, more than 1 million cases and 50,000 deaths occur worldwide [2], and disease burden is estimated at 2.9 million disability adjusted life years (DALYs) [3]. Approximately 5–10% of symptomatic patients develop severe manifestations, including multi-system dysfunction (historically referred to as Weil’s disease), and 15% of these may die [1, 4]. Antimicrobial therapy initiated within 7 days of disease onset may shorten duration of illness and improve survival [5, 6]. However, non-specific presentations, varying from undifferentiated fever to aseptic meningitis or pulmonary hemorrhage [7], and clinical uncertainty relative to similar illnesses (e.g., dengue, malaria, enteric fever, typhus, and viral hepatitis) can lead to delayed diagnosis and intervention [8–10]. Consequently, clinicians worldwide need accurate, reliable and rapid diagnostic tests for leptospirosis.

The legacy gold standards for diagnosing leptospirosis, the microscopic agglutination test (MAT) and hemoculture, have limitations. MAT requires maintenance of reference *Leptospira* cultures and paired sera for diagnosis, and blood cultures are generally low yield. While expanding, molecular techniques are often inaccessible in emergency health units where leptospirosis patients typically present and their sensitivity declines within a few days after disease onset, concomitantly with waning bacteremia [11, 12]. Other serological assays generally have inadequate sensitivity in the early phase of infection [13], and combining techniques can boost acute-phase detection [14, 15]. Nonetheless, the development of a single platform having adequate discriminatory capacity during acute illness and sufficient portability for bedside or field use has been thus far intangible [16].

The Dual Path Platform (DPP) (Chembio Diagnostic Systems, Medford, New York, USA) utilizes a variation of lateral flow technology, whereby the biological sample and the colorimetric marker are separately delivered on perpendicular nitrocellulose membranes. High concentrations of recombinant leptospiral immunoglobulin-like (rLig) proteins serve as antigens. We previously demonstrated the assay’s sensitivity on acute-phase sera, 78–85% for hospitalized patients, which was similar to a widely used IgM-ELISA [17]. Specificity was ≥95% among sera from clinically compatible illnesses and 86% among healthy slum dwellers. Based on these serum data, the Brazilian Ministry of Health regulatory department responsible for approval and supervision of pharmaceuticals, health services, and medical devices, approved the DPP for diagnosing human leptospirosis in 2011. However, DPP accuracy using finger stick blood (FSB) has not been previously evaluated. Herein, we present findings from a prospective clinical study, aiming to evaluate FSB DPP performance compared to: 1) venous whole blood (VWB) and serum DPP, 2) serum IgM-ELISA, and 3) clinical impression alone. Additionally, we measured DPP reproducibility and clinical utility.

## Methods

From April 18 to October 18, 2012, we sequentially recruited patients from Hospital Couto Maia (HCM), a reference infectious diseases hospital in Salvador, Brazil, where the health system assists ~90% of the regional leptospirosis cases requiring hospitalization. We aimed to enroll 196 confirmed leptospirosis patients. We calculated the sample size using a desired 95% confidence interval precision of ±10% around the anticipated acute-phase sensitivity of 85% [17].

### Ethics statement

We obtained written informed consent for all patients per protocols approved by Oswaldo Cruz Foundation, HCM, and Yale University. For minors and mentally altered adults, we obtained written consent from a legal representative.

### Inclusion and exclusion

Physicians routinely assign provisional diagnoses at hospital admission using clinical and basic laboratory assessments. Daily, we reviewed the assigned diagnoses to triage patients with suspected diseases clinically compatible with leptospirosis for study inclusion (S1 Table). Among triaged patients, we enrolled those presenting with acute fever (≥38°C or per history) and ≥1 of the following: acute renal failure (creatinine >1.5 mg/dL or oliguria); jaundice; acute hepatitis (AST or ALT >75 and <3,000 U/L; or total bilirubin >3 mg/dL); spontaneous hemorrhage; enteric fever (i.e., syndrome of diarrhea/constipation associated with fever and abdominal pain); bilateral conjunctival suffusion; aseptic meningitis; or undifferentiated fever. We included aseptic meningitis patients that had non-turbid, non-purulent cerebrospinal fluid (CSF) containing 10–2,000 cells/m^3^, ≤150 mg/dL protein, ≥40 mg/dL glucose, and negative results for bacterial meningitis on the bacterioscopic or latex exam. We sought aseptic meningitis likely to elicit clinical suspicion for leptospirosis at onset, and therefore mandated ≥1 epidemiologic risk factor ≤30 days of onset for inclusion: 1) floodwater, sewer water, or mud contact, 2) rats sighted at home or work, or 3) domiciled or working in a high-risk environment (i.e., slum community or animal farm). We excluded patients <5 years due to low relative risk, and those unavailable for clinical evaluation secondary to death or discharge.

### Data collection

At enrollment, we obtained demographic, epidemiological, and clinical data. We also recorded daily clinical evolution during hospitalization, including final diagnostic impression and survival. The hospital team performed laboratory tests, except reference diagnostics for leptospirosis outlined below, at its discretion without our input. One of us (SAN), clinically trained in internal medicine and pediatrics, performed an un-blinded, comprehensive records review at discharge or death to ascertain the most probable final diagnosis of enrollees without MAT- or culture-confirmed leptospirosis. He also classified final diagnoses as either primarily clinical or supported by laboratory diagnostics.

### Specimens

We evaluated three specimen types: 1) FSB, 2) VWB in EDTA, and 3) serum at enrollment. Sera remained at -20°C until diagnostic testing by MAT, IgM-ELISA (whole-*Leptospira* IgM-ELISA; Bio-Manguinhos, Rio de Janeiro, Brazil [15]), and DPP (Bio-Manguinhos, Rio de Janeiro, Brazil). We doggedly pursued convalescent serum collection (generally 15–30 days after admission) primarily via home visits. Only refusal, death, or inability to contact the patient after ≥3 attempts justified incomplete convalescent serum collection.

### Case confirmation by reference tests

We aimed for completeness of acute-phase EMJH blood culture collection and MAT analysis with paired acute- and convalescent-phase sera. Confirmed leptospirosis included 1) *Leptospira* blood culture growth, or 2) reactive MAT by ≥1 criterion: (i) ≥4-fold rise in titer between acute- and convalescent-phase sera, (ii) seroconversion (undetectable acute-phase titer and convalescent-phase titer ≥1:200), or (iii) sample titer ≥1:800. We defined probable leptospirosis by an MAT titer of 1:200 or 1:400 where confirmation criteria remained unsatisfied. The MAT panel included 10 *Leptospira* strains representing eight serovars and eight serogroups [17].

### DPP evaluation

For prospectively enrolled patient samples, we performed the DPP per manufacturer instructions in two stages: 1) point-of-care FSB and VWB assays at enrollment, and 2) batched serum assays in a controlled laboratory environment. We informed treating physicians of the experimental whole blood results without an associated recommendation for clinical management. Methodological nuances by specimen type follow.

#### Whole blood

For FSB, we punctured one ventral finger pad by lancet for ~10 μL of capillary blood applied immediately to the DPP at bedside. For VWB, we filled one 4-mL vacutainer tube containing EDTA and aliquoted 10 μL by capillary tube to the DPP within 2 hours. We interpreted each at 20 minutes.

#### Serum

We performed a blinded DPP evaluation of acute-phase sera from prospectively enrolled patients. A statistician assigned a randomized unique code to each specimen to ensure sample anonymity. We stored sera at -20°C until assay, at which time we used 5 μL of serum, applied buffer, and interpreted results at 20 minutes [17].

#### Visual interpretation

Three operators independently interpreted DPP results using a standardized visual guide. The dichotomous result (i.e., positive or negative) was determined by concordance of ≥2 visual interpreters. Generally, two of the operators consisted of the same researchers who clinically evaluated the patient for inclusion criteria. We blinded all operators, however, to all confirmatory diagnostics, and all operators for the serum evaluation additionally remained blinded to clinical data.

#### Statistical analyses

We used REDCap [18] for data management and SAS 9.2 (SAS Inst.; Cary, NC, USA) to compare diagnostic performance of the DPP using FSB to that of DPP using VWB and serum, serum IgM-ELISA, and clinical impression alone. To limit misclassification, we excluded two enrollees with probable leptospirosis by the MAT definition. Similarly, we excluded two records with indeterminate IgM-ELISA results from the ELISA-specific calculations. Notably, one confirmed leptospirosis case did not have FSB sampling due to precipitous death upon arrival to hospital and we excluded this patient from FSB measures of diagnostic performance. Otherwise, we had no missing DPP or reference standard data.

Primary outcomes were sensitivity, specificity, predictive values, and likelihood ratios for the FSB DPP at enrollment and were evaluated according to STARD principles [19]. We also estimated these parameters for the DPP using the other specimen types, for IgM-ELISA, and for clinical impression. The accuracy parameters were estimated separately for two subgroups of patients defined by clinical syndrome (i.e., aseptic meningitis versus all other syndromes combined). For simplicity, we heretofore refer to the combination of all other syndromes compatible with acute clinical leptospirosis as *classic leptospirosis*. We focused our results and discussion on this classic leptospirosis group, as it most represents the most common clinical presentations of severe leptospirosis. We measured FSB DPP clinical utility with correlates of medical management, namely initiation of antibiotic therapy and length of hospitalization. Too few deaths occurred for meaningful comparison of survival.

We calculated 95% Exact binomial Clopper-Pearson confidence limits (2-tailed Z, α = 0.05) for frequencies. We tested differences in binomial proportions using the Fisher’s exact test (2-tailed, α = 0.05) and tested mean differences using the unpaired t-test (2-tailed t, α = 0.05) between confirmed and not confirmed enrollees. We did not employ hypothesis testing to measures of clinical utility (i.e., length of hospitalization, antibiotic use) due to inadequate power. We empirically assigned numeric pre-test probabilities for a definitive diagnosis of leptospirosis at three levels (i.e., low [20%], moderate [50%], and high [80%]), and applied the consequent likelihood ratios (LR) for each type of diagnostic approach evaluated (i.e., FSB DPP, VWB DPP, serum DPP, serum IgM-ELISA, and clinical impression alone) to estimate post-test probabilities. The low, moderate, and high probability groups represent three distinct clinical diagnostic challenges encountered in this study population and parallel usual decision-making that clinicians encounter in the tropics. The high probability group approximated patients with acute fever, renal failure, and jaundice (i.e., Weil’s disease) (18 patients; 14 [78%] MAT-confirmed). Moderate probability roughly correlated to all enrollees suspected to have leptospirosis by triage physicians (45 patients; 27 [60%] MAT-confirmed), and low probability represented aseptic meningitis patients with an epidemiological exposure for leptospirosis (22 patients; 5 [23%] MAT-confirmed). Finally, we measured kappa inter-operator reproducibility by specimen type.

## Results

### Enrollee characteristics

**Fig 1:**
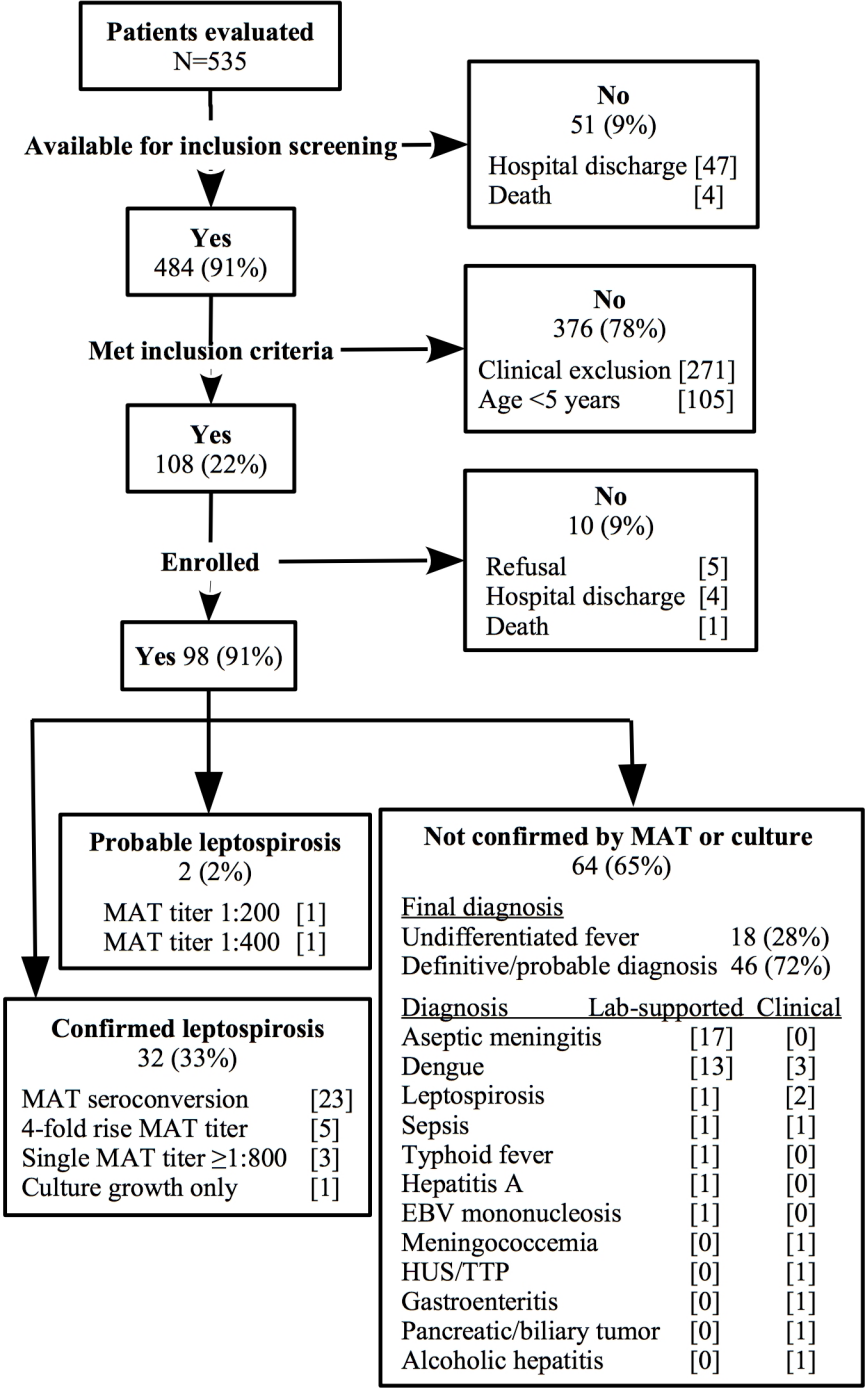
Recruitment of patients hospitalized for acute febrile illness compatible with leptospirosis–Salvador, Brazil 2012. * Undifferentiated fever includes any other febrile illness consistent with acute clinical leptospirosis not elsewhere categorized. EBV = Epstein-Barr virus. HUS/TTP = hemolytic uremic syndrome/thrombotic thrombocytopenic purpura.

We identified 535 patients with leptospirosis-compatible illnesses and we could screen 484 (91%) for study inclusion. Of the screened patients, 108 (22%) met inclusion criteria, and we enrolled 98 (91%) of these (Fig 1). Among the enrollees, the most common triage diagnoses were leptospirosis (n = 46, 47%), dengue (16, 16%), and aseptic meningitis (22, 22%). We successfully paired acute and convalescent-phase sera for 68 (69%) enrollees. Paired sera status occurred among not confirmed enrollees (77%) more frequently than confirmed leptospirosis (59%) (Table 1), reflecting intensive efforts to accurately classify disease status with convalescent specimens. We confirmed 32 (33%) leptospirosis cases. Four cases had a positive hemoculture, three of which we also confirmed by seroconversion. Only one was confirmed by hemoculture alone. Positive cultures demonstrated serogroup Icterohaemorrhagiae, sorovar Copenhageni for the four isolates. Notably, five (23%) of 22 aseptic meningitis enrollees were confirmed leptospirosis. Of 64 patients not confirmed as leptospirosis, we considered three at discharge to have possible leptospirosis; we based one assignment on acute-phase IgM-ELISA at a reference laboratory and the other two on clinical manifestations and biochemical lab results highly consistent with acute clinical leptospirosis (Fig 1).

**Table 1:** Characteristics of 96 enrolled patients hospitalized with acute febrile illness compatible with leptospirosis–Salvador, Brazil 2012.

Category	Characteristic	Confirmed Leptospirosis (N = 32)		Not Confirmed (N = 64)		P Value
		N	n (%) or mean ± SD	N	n (%) or mean ± SD	
Demographics	Age in years	32	40.4 ± 15.9	63	28.2 ± 15.3	<0.001
	Male sex	32	28 (88)	63	39 (62)	0.04
Clinical presentation	Days of symptoms	32	6.6 ± 2.7	63	8.2 ± 9.0	0.33
	Antibiotic prior to arrival	25	10 (40)	53	13 (25)	0.31
	Jaundice	32	22 (69)	63	14 (22)	<0.001
	Acute renal failure	32	24 (75)	64	15 (23)	<0.001
	Hemoptysis	32	10 (31)	63	10 (16)	0.06
	Renal failure and jaundice*	32	14 (44)	63	4 (6)	<0.001
Laboratories at hospitalization	Platelets (/μL)	32	133 ± 96	62	178 ± 133	0.09
	Creatinine (mg/dL)	32	3.6 ± 2.9	57	1.7 ± 2.1	<0.001
	Bilirubin, total (mg/dL)	22	11.3 ± 9.8	34	3.4 ± 8.5	0.002
	Alanine transaminase (U/L)	31	99 ± 84	56	214 ± 398	0.12
Leptospirosis as admitting clinical impression		32	26 (81)	64	20 (31)	<0.001
Paired acute and convalescent sera		32	19 (59)	64	49 (77)	0.05
Clinical course	Days of hospitalization	32	10.1 ± 5.3	63	7.0 ± 6.8	0.03
	Admitted to intensive care	32	4 (13)	62	4 (6)	0.27
	Death	32	1 (3)	64	4 (6)	0.63

SD = standard deviation. P value for t-test (continuous variables) or Fisher’s exact test (binomial proportions).

* Proxy for Weil’s disease.

Leptospirosis confirmed patients were older (mean 40 versus 28 years, respectively, P <0.001), more frequently male (88% versus 62%, respectively, P = 0.04), and more ill as measured by jaundice (69% versus 22%, respectively, P <0.001) and renal failure (75% versus 23%, respectively, P <0.001). They were also hospitalized longer (mean 10 versus 7 days, respectively, P = 0.06), more frequently required intensive care (13% versus 6%, P = 0.27), and more commonly designated leptospirosis at triage (81% versus 31%, respectively, P <0.001). Deaths were infrequent among confirmed and not confirmed cases (5% overall).

### Diagnostic accuracy

#### Sensitivity and specificity

DPP sensitivity for classic leptospirosis was 93% by FSB and 96% by VWB (Table 2). Both POC assays were more sensitive than serum DPP (85%) and serum IgM-ELISA (81%), and similar to clinical impression (96%). The FSB and the VWB DPP detected 40% (2 of 5) and 80% (4 of 5) of the confirmed leptospiral meningitis, respectively, none of which clinicians suspected to be leptospirosis at triage. DPP specificity for classic leptospirosis was 80% by FSB and 75% by VWB (Table 2); it was higher for serum DPP (87%) and IgM-ELISA (100%), but poor for clinical impression (57%). Twelve not confirmed, classic leptospirosis patients lacked convalescent-phase sera and for these we could not completely rule out leptospirosis. Therefore, we calculated specificity for sub-groups of FSB DPP samples, comparing 35 classic leptospirosis patients with paired sera (86%, 95% CI 70–95%) to 11 classic leptospirosis patients with only acute-phase sera (64%, 95% CI 31–89%). The results show greater diagnostic uncertainty among single-sera enrollees. For meningitis patients, specificity was 82% by FSB DPP and 88% by VWB DPP; it was higher for serum DPP (94%), IgM-ELISA (94%) and clinical impression (100%).

**Table 2:** Comparative acute-phase sensitivity, specificity, and predictive values for diagnosing leptospirosis by DPP assay using finger stick blood (FSB), venous whole blood (VWB), and serum, by serum IgM-ELISA, and by clinical impression for 96 patients–Salvador, Brazil 2012.

Test/Specimen type by patient group	Pos/Conf	Se (95% CI)	Neg/Not conf	Sp (95% CI)	PPV (95% CI)	NPV (95% CI)
***Classic leptospirosis***						
DPP/FSB	25/27	92.6 (75.7–99.1)	37/46	80.4 (66.1–90.6)	73.5 (60.5–83.4)	94.9 (82.9–98.6)
DPP/VWB	26/27	96.3 (81.0–99.4)	35/47	74.5 (59.6–86.0)	68.4 (51.3–82.5)	97.2 (85.4–99.5)
DPP/Serum	23/27	85.2 (66.3–95.7)	41/47	87.2 (74.2–95.1)	79.3 (60.3–92.0)	91.1 (78.8–97.5)
IgM-ELISA/Serum	21/26	80.8 (60.7–93.5)	46/46	100.0 (92.2–100.0)	100.0 (83.9–100.0)	90.2 (78.6–96.7)
Clinical impression	26/27	96.3 (81.0–99.4)	27/47	57.4 (42.2–71.7)	56.5 (41.1–71.1)	96.4 (81.6–99.4)
***Meningitis***						
DPP/FSB	2/5	40.0 (5.3–85.3)	14/17	82.4 (56.6–96.2)	40.0 (6.5–84.6)	82.4 (56.6–96.0)
DPP/VWB	4/5	80.0 (28.4–99.5)	15/17	88.2 (63.6–98.5)	66.7 (22.7–94.7)	93.8 (69.7–99.0)
DPP/Serum	3/5	60.0 (14.7–94.7)	16/17	94.1 (71.2–99.0)	75.0 (20.3–95.9)	88.9 (65.2–98.3)
IgM-ELISA/Serum	4/5	80.0 (28.4–99.5)	16/17	94.1 (71.2–99.0)	80.0 (28.8–96.7)	94.1 (71.2–99.0)
Clinical impression	0/5	0.0 (0.0–52.2)	17/17	100.0 (80.5–100.0)	Undefined	77.3 (54.6–92.1)

Pos = positive test result; Neg = negative test result; Conf = confirmed leptospirosis; Not conf = not confirmed for leptospirosis; Se = sensitivity; Sp = specificity; CI = confidence interval; PPV = positive predictive value; NPV = negative predictive value; FSB = Finger stick blood; VWB = venous whole blood.

#### Predictive values and likelihood ratios

For classic leptospirosis, PPV was acceptable for FSB DPP (74%), VWB DPP (68%) and serum DPP (79%), and exceptional for IgM-ELISA (100%) (Table 2). Clinical impression conferred poor PPV (57%). NPV did not differ between FSB DPP, VWB DPP and clinical impression (95–97%); although, NPV was lower for both serum DPP (91%) and IgM-ELISA (90%).

For classic leptospirosis, the FSB DPP and serum DPP each demonstrated moderate positive LRs (4.7 and 6.7, respectively) (Table 3). The FSB DPP however showed a high-impact negative LR (0.09), while the serum DPP negative LR (0.17) was moderate. The VWB DPP and clinical impression had both the lowest positive LR (3.8 and 2.3, respectively) and stronger negative LRs (0.05 and 0.06, respectively). The IgM-ELISA positive LR (>37) was excellent and it had a negative LR of 0.20. Sample size limited interpretation of diagnostic accuracy for meningitis (Tables 2 and 3).

**Table 3:** Comparative acute-phase diagnostic likelihood ratios and post-test probabilities for accurately detecting human leptospirosis by DPP assay using finger stick blood (FSB), venous whole blood (VWB), and serum, by serum IgM-ELISA, and by clinical impression for 96 patients–Salvador, Brazil 2012.

Test/Specimen type by patient group	Positive LR (95% CI)	Positive Post-TP, by level of Pre-TP			Negative LR (95% CI)	Negative Post-TP, by level of Pre-TP		
		L	M	H		L	M	H
***Classic leptospirosis***								
DPP/FSB	4.73 (2.61–8.59)	54.2	82.5	95.0	0.09 (0.02–0.35)	2.2	8.3	26.5
DPP/VWB	3.77 (2.30–6.18)	48.5	79.0	93.8	0.05 (0.05–0.34)	1.2	4.8	16.7
DPP/Serum	6.67 (3.11–14.32)	62.5	87.0	96.4	0.17 (0.07–0.42)	4.1	14.5	40.5
IgM-ELISA/Serum	37.15 *(5.30–260.52)	90.3	97.4	99.3	0.20 (0.09–0.43)	4.7	16.5	44.1
Clinical impression	2.26 (1.61–3.18)	36.1	69.3	90.0	0.06 (0.01–0.45)	2.5	5.7	19.4
***Meningitis***								
DPP/FSB	2.27 (0.51–10.01)	36.2	69.4	90.1	0.73 (0.34–1.54)	15.4	42.2	74.5
DPP/VWB	6.80 (1.72–26.86)	63.0	87.2	96.5	0.23 (0.04–1.32)	5.4	18.7	47.9
DPP/Serum	9.60 (1.26–72.96)	70.6	90.6	97.5	0.43 (0.15–1.26)	9.7	30.1	63.2
IgM-ELISA/Serum	13.60 (1.93–95.71)	77.3	93.2	98.2	0.21 (0.04–1.23)	5.0	17.4	45.7

DPP = Dual Path Platform; CI = confidence interval; LR = likelihood ratio; Post-TP = post-test probability; VWB = venous whole blood; ELISA = enzyme-linked immunosorbent assay; Pre-TP = pre-test probability; the clinical Pre-TP of leptospirosis was designated as low, moderate, or high; L = low clinical Pre-TP of leptospirosis (20%); M = moderate clinical Pre-TP (50%); H = high clinical Pre-TP (80%); NA = not applicable; Undefined = contains operation divisible by zero. Values not calculated for clinical impression among meningitis patients.

*Positive LR underestimates the true value, as 45 was used as number of true negatives and one was used as false negative in order to avoid division by zero.

#### False-positive FSB DPP

Of the nine non-meningitis patients with false-positive FSB DPP at enrollment, two (22%) had leptospirosis as the most probable diagnosis upon final records review based on the constellation of clinical manifestations and basic laboratory results. We classified another three patients as undifferentiated fever. The remainder had reasonable evidence of an alternative disease: one dengue fever by IgM ELISA; one hepatitis A (versus dengue) by IgM ELISA for both hepatitis A and dengue; one hemolytic uremic syndrome/thrombotic thrombocytopenic purpura (HUS/TTP) by clinical laboratory profile; and one rat bite fever bacteremia in a patient who reared laboratory rats.

### Clinical utility

For confirmed leptospirosis with a positive FSB DPP, length of hospitalization appeared greater than for confirmed patients with a negative FSB DPP (11.1 ±5.1 days versus 4.8 ±2.3 days for positive and negative FSB DPP, respectively), as did frequency of antibiotic administration (93% versus 80%, respectively). Among not confirmed patients, we saw no observable difference in length of hospital stay (7.0 ±4.4 days versus 7.1 ±7.3 days for positive and negative FSB DPP, respectively), but we found a higher proportion of antibiotic administration (64% versus 47%, respectively). All clinically suspected leptospirosis patients received antibiotic therapy while in hospital, regardless of FSB DPP result. By clinical review, one aseptic meningitis patient, who we later confirmed as leptospirosis, received antibiotics in response to the FSB DPP result.

### Reproducibility

Kappa inter-operator agreement for FSB DPP (87%, 95% CI 78–97%) and serum (87%, 95% CI 76–97%) was very good; it was excellent for VWB (96%, 95% CI 90–100%).

## Discussion

Our findings indicate that the DPP assay is portable, accurate, and reliable for early diagnosis of human leptospirosis using FSB, VWB and serum. Among clinical syndromes most consistent with classic leptospirosis in our patient sample (i.e., non-meningitis), FSB DPP showed equivalent accuracy to serum DPP. This removes field serum preparation from the diagnostic process and establishes the DPP as an ideal diagnostic tool for leptospirosis [16]. While not directly compared, the DPP showed superior sensitivity and similar specificity to the reported accuracy for three peer rapid assays for leptospirosis in a prospective study with sera from the Netherlands [13]. In that evaluation, the sensitivity using the initial acute-phase specimen was 51–69% and the specificity was 96–98%. Another contemporary rapid assay had shown results similarly promising to the DPP using a lower MAT threshold for single-titer case definition (i.e., 1:400 versus 1:800 for DPP) among French Polynesian sera [20]. However, the investigators did not evaluate their performance with bedside blood samples.

Because we deliberately enrolled patients with a variety of syndromes to represent the largely protean manifestations of leptospirosis, clinical recognition of the disease was more difficult than it would have been if we had enrolled only those patients with the classical presentations of severe leptospirosis (i.e., Weil’s disease). Therefore, as expected, DPP proved superior to the clinical impression of the experienced infectious disease physicians from this reference center. Nevertheless, the referral process for hospitalization at HCM selects for more severely ill patients. We previously showed that serum DPP performance correlates with disease severity [17], and this association may also be true for the whole blood specimens used in this assessment.

In subgroup analysis of patients classified as false positive by FSB DPP, we identified two cases that were highly consistent with acute clinical leptospirosis but lacked confirmation with convalescent-phase sera. If these were indeed misclassified, the point estimate for FSB DPP specificity among classic leptospirosis cases after removing them from the group of not confirmed patients would change from 80% to 84% (95% CI 70–93%). The latter result would be more consistent with specificity previously calculated among high-risk slum residents in a large laboratory-based evaluation of the serum DPP [17].

Interestingly, we confirmed an impressive 23% of aseptic meningitis enrollees with legitimate history of a risk exposure to have leptospirosis. This suggests that primary neuroleptospirosis may be more common than previously appreciated in this epidemiological setting. Our findings further highlight the usefulness of the DPP in diagnosing leptospiral meningitis, a clinical expression of leptospirosis well recognized but less commonly detected [21, 22].

We examined diagnostic performance for two whole blood specimens easily obtained in most peripheral health centers and we focused on the simplest, the bedside FSB DPP. It is nonetheless important to note that the VWB DPP correctly detected three more patients than the FSB assay, but this was at the expense of specificity. This observation needs replication; however, plausible explanations include an EDTA-induced reduction of antibody or complement complex formation [23, 24], differences in the biochemical profile of venous and capillary samples, and differential lighting between the laboratory and sunlit hospital wards potentially modifying visual interpretation. Regardless, VWB remains an alternative to FSB in certain circumstances (i.e., lancets unavailable). In comparison to serum DPP and IgM-ELISA, both FSB and VWB DPP assays showed better sensitivity. The FSB DPP specificity and PPV were inferior, however, to those for IgM-ELISA in this patient group. While no patient with a false-positive FSB DPP reported a prior diagnosis of leptospirosis, it is possible that prior *Leptospira* infection in this largely at-risk patient group may have contributed to the false result.

When clinical suspicion for leptospirosis was high, the positive post-test probability for all DPP samples was highly satisfactory; similarly, the negative post-test probability for all DPP samples showed excellent discriminatory effect. In the clinical scenario where diagnostic testing is most impactful on clinical decision-making (i.e., moderate pre-test clinical suspicion of leptospirosis), the IgM-ELISA demonstrated perhaps the most favorable profile for detecting true leptospirosis cases. In this context, the IgM-ELISA showed a positive post-test probability of 97% compared to 83% for the FSB DPP. Conversely, in the same scenario, the FSB DPP more accurately ruled out leptospirosis with a negative post-test probability of 8% compared to 17% for IgM-ELISA. Nonetheless, patients for whom clinicians have moderate to high clinical suspicion for leptospirosis and the FSB DPP is negative may benefit from confirmatory testing where accessible.

We observed that confirmed leptospirosis patients with a positive DPP result remained in hospital longer than those with negative DPP results. We also found increased use of antibiotics by patients who had a positive DPP result, even though the difference was not statistically significant. However, we did not design this study to measure clinical utility. This should be robustly evaluated once the DPP is routinely implemented. Other study limitations include the geographically limited sample and the inability to fully blind operators to clinical data for acute-phase FSB and VWB DPP interpretation. Due to an unusually mild epidemic season, we enrolled a significantly smaller number of confirmed leptospirosis patients than anticipated. As a result, small sample size limits our conclusions related to confirmed leptospirosis presenting with aseptic meningitis and other subpopulation analyses. It also limits the precision of the primary outcome. Lastly, incomplete convalescent sampling may underestimate FSB DPP specificity because we were unable to confirm all leptospirosis cases using the gold standard diagnostic test.

In summary, the FSB DPP is a rapid, portable alternative to laboratory-based diagnostics for the detection of severe leptospirosis. It expands the diagnostic landscape for effective clinical and outbreak management, and may improve detection of leptospirosis cases presenting with meningitis. Next generation POC assays may improve specificity by distinguishing IgM from IgG, particularly in endemic regions where prior exposures to *Leptospira* may affect immunoglobulin detection [25, 26], and utilizing additional molecular targets [27, 28]. The DPP warrants further investigation in broader epidemiological settings, in direct comparison to peer rapid serological assays, and in diagnosing mild and atypical presentations.

## Supporting information

S1 Table Admitting diagnoses that may mimic acute clinical leptospirosis and which prompted inclusion screening for 535 hospitalized patients by the study team–Salvador, Brazil 2012. * Common admitting diagnosis at study site. ** SIRS, endocarditis and myocarditis are syndromes that may have non-bacterial and non-viral etiologies. NOS = not otherwise specified.
(DOCX)

S2 Table STARD checklist for the prospective study of the diagnostic accuracy for the Dual Path Platform (DPP) for human leptospirosis–Salvador, Brazil 2012. (DOCX)

S1 File Data dictionary for the prospective study of the diagnostic accuracy for the Dual Path Platform (DPP) for human leptospirosis–Salvador, Brazil 2012. (DOC)

S1 Dataset Data files for the prospective study of the diagnostic accuracy for the Dual Path Platform (DPP) for human leptospirosis–Salvador, Brazil 2012. (XLSX)
