# Supplementary material for: Prospective evaluation of accuracy and clinical utility of the Dual Path Platform (DPP) assay for the point-of-care diagnosis of leptospirosis in hospitalized patients
Source: PLoS Negl Trop Dis. 2018 Feb 20;12(2):e0006285. doi: 10.1371/journal.pntd.0006285 (PMC5834199; doi:10.1371/journal.pntd.0006285)
Supplement: S1 Table — * Common admitting diagnosis at study site. ** SIRS, endocarditis and myocarditis are syndromes that may have non-bacterial and non-viral etiologies. NOS = not otherwise specified. (DOCX) [file pntd.0006285.s001.docx]

**S1 Table.** Admitting diagnoses that may mimic acute clinical leptospirosis and which prompted inclusion screening for 535 hospitalized patients by the study team – Salvador, Brazil 2012.

| **Bacterial** | **Viral** | **Parasitic** |
| --- | --- | --- |
| Acute bacterial illness, NOS | Acute viral illness, NOS * | Malaria |
| Brazilian spotted fever and other rickettsial diseases | Aseptic (viral) meningitis * | Schistosomiasis |
| Brucellosis | Dengue (classic or hemorrhagic) * | Visceral leishmaniasis |
| Cholangitis | Hantavirus |  |
| Cholecystitis, cholodocholithiasis | Hepatitis, any acute * |  |
| Endocarditis ** | Influenza * |  |
| Leptospirosis * | Myocarditis ** |  |
| Meningococcal disease | Yellow fever |  |
| Pyelonephritis |  |  |
| Systemic inflammatory response syndrome (SIRS) *, ** |  |  |
| Typhoid/enteric fever * |  |  |

* Common admitting diagnosis at study site. ** SIRS, endocarditis and myocarditis may have non-bacterial and non-viral etiologies. NOS = not otherwise specified.
