# Supplementary material for: Prospective evaluation of accuracy and clinical utility of the Dual Path Platform (DPP) assay for the point-of-care diagnosis of leptospirosis in hospitalized patients
Source: PLoS Negl Trop Dis. 2018 Feb 20;12(2):e0006285. doi: 10.1371/journal.pntd.0006285 (PMC5834199; doi:10.1371/journal.pntd.0006285)
Supplement: S1 File — (DOC) [file pntd.0006285.s003.doc]

#### S1 File – Data dictionary for the prospective study of the diagnostic accuracy for the Dual Path Plaform (DPP) for human leptospirosis – Salvador, Brazil 2012.

#### Table *DPP_Inclusion (N=535)*

#### *Inclusion criteria and status for evaluated patients*

| **VARIABLE** | **Field Type** | Format/Value | **Special Info** | **PROMPT** |
| --- | --- | --- | --- | --- |
| ID | NUMBER | #### |  | ID Number |
| SUSPDIAG | CHAR | $30 |  | Suspected diagnosis by clinician |
| CRITINCL | CHAR | $8 | “Sim” (Yes), “Nao” (No), “Não sabe” (Unknown) | Patient met inclusion criteria? |
| CRITNAO | CHAR | $255 |  | If patient did not meet inclusion criteria, why not? |
| INCLUIDO | CHAR | $12 | “Incluido” (Enrolled), “Não incluido” (Not enrolled) | Patient enrolled in study? |
| INCLNAO | CHAR | $9 | “Criterios” (Did not meet criteria), “Alta” (Discharge), “Idade” (Age), “Obito” (Death) | If patient not enrolled, why not? |
| SINDLEPTO | CHAR | $3 | “Sim” (Yes), “Nao” (No) | Clinically suspected leptospirosis |
| SINDIRA | CHAR | $3 | “Sim” (Yes), “Nao” (No) | Syndrome of acute renal insufficiency (Cr>1.5 e/ou oliguria) |
| SINDFIG | CHAR | $3 | “Sim” (Yes), “Nao” (No) | Syndrome of liver dysfunction (TBili>3 e/ou ALT/AST>75) |
| SINDSUFUS | CHAR | $3 | “Sim” (Yes), “Nao” (No) | Syndrome of bilateral conjunctival suffusion |
| SINDICT | CHAR | $3 | “Sim” (Yes), “Nao” (No) | Syndrome of jaundice |
| SINDHEMOR | CHAR | $3 | “Sim” (Yes), “Nao” (No) | Syndrome of hemorrhage |
| SINDFEBENT | CHAR | $3 | “Sim” (Yes), “Nao” (No) | Syndrome of enteric fever |
| SINDFEBINDIF | CHAR | $3 | “Sim” (Yes), “Nao” (No) | Syndrome of undifferentiated fever (if does not fit any other syndrome category) |
| SINDMENING | CHAR | $3 | “Sim” (Yes), “Nao” (No) | Syndrome of aseptic meningitis |

#### Table *DPP_V01DPP_AlternateDiagnoses (N=41)*

#### *Most likely final diagnosis for enrollees Not Confirmed for leptospirosis*

#### Note: Some records missing due to unavailability of medical charts

| **VARIABLE** | **Field Type** | Format/Value | **Special Info** | **PROMPT** |
| --- | --- | --- | --- | --- |
| 1.1ID | NUMBER | #### |  | ID number |
| 2.1ELEP | NUMBER | # | 1=Positive; 0=Negative; 9=Not done | IgM ELISA for leptospirosis |
| 2.2DIGM | NUMBER | # | 1=Positive; 0=Negative; 9=Not done | IgM ELISA for dengue |
| 2.3DIGG | NUMBER | # | 1=Positive; 0=Negative; 9=Not done | IgG ELISA for dengue |
| 2.4DNS | NUMBER | # | 1=Positive; 0=Negative; 9=Not done | NS1 for dengue |
| 2.5DPCR | NUMBER | # | 1=Positive; 0=Negative; 9=Not done | PCR for dengue |
| 2.6HIVTR | NUMBER | # | 1=Positive; 0=Negative; 9=Not done | HIV rapid test |
| 2.7HIVWB | NUMBER | # | 1=Positive; 0=Negative; 9=Not done | HIV Western Blot |
| 2.8HAIGM | NUMBER | # | 1=Positive; 0=Negative; 9=Not done | IgM Hepatitis A |
| 2.9HAIGG | NUMBER | # | 1=Positive; 0=Negative; 9=Not done | IgG Hepatitis A |
| 2.10HBSAG | NUMBER | # | 1=Positive; 0=Negative; 9=Not done | HBsAg Hepatitis B |
| 2.11HBEAG | NUMBER | # | 1=Positive; 0=Negative; 9=Not done | HBeAg Hepatitis B |
| 2.12HBIGM | NUMBER | # | 1=Positive; 0=Negative; 9=Not done | Anti-HBsAg IgM Hepatitis B |
| 2.13HBIGG | NUMBER | # | 1=Positive; 0=Negative; 9=Not done | Anti-HBsAg IgG Hepatitis B |
| 2.14HBCAG | NUMBER | # | 1=Positive; 0=Negative; 9=Not done | HBcAg Hepatitis B |
| 2.15HCULTA | NUMBER | # | 1=Positive; 0=Negative; 9=Not done | Aerobic blood culture |
| HCULTAI | CHAR | $25 | If HCULTA=1 | Aerobic culture isolate |
| 2.16HCULTAN | NUMBER | # | 1=Positive; 0=Negative; 9=Not done | Anaerobic blood culture |
| HCULTANI | CHAR | $25 | If HCULTAN=1 | Anaerobic culture isolate |
| 2.17FCULT | NUMBER | # | 1=Positive; 0=Negative; 9=Not done | Stool culture |
| 2.18UCULT | NUMBER | # | 1=Positive; 0=Negative; 9=Not done | Urine culture |
| 2.19OTST1 | NUMBER | # | 1=Positive; 0=Negative; 9=Not done | Other test #1 result |
| OTST1N | CHAR | $255 |  | Name of other test #1 |
| 2.20OTST2 | NUMBER | # | 1=Positive; 0=Negative; 9=Not done | Other test #2 result |
| OTST2N | CHAR | $255 |  | Name of other test #2 |
| 2.20AOTST3 | NUMBER | # | 1=Positive; 0=Negative; 9=Not done | Other test #3 result |
| OTST3N | CHAR | $255 |  | Name of other test #3 |
| 2.20BOTST4 | NUMBER | # | 1=Positive; 0=Negative; 9=Not done | Other test #4 result |
| OTST4N | CHAR | $255 |  | Name other test #4 |
| 2.20COTST5 | NUMBER | # | 1=Positive; 0=Negative; 9=Not done | Other test #5 result |
| OTST5N | CHAR | $255 |  | Name of other test #5 |
| 3.1DGALT | NUMBER | # | 1=Yes; 2=No; 9=Insufficient information | Alternative diagnosis more likely than leptospirosis? |
| 3.2PLEPT | NUMBER | # | 1=High; 2=Moderate; 3=Low | Clinical probability of leptospirosis per final study team review |
| 3.3DGFIN | NUMBER | # | 1=Lepto; 2=Dengue; 3=Hepatitis; 4=Typhoid, 5=Other; 9=Unknown | Final diagnosis (suspected or confirmed) per study team review |
| 3.4DFOUT | CHAR | $255 | If DGFIN=5 | Other final diagnosis |
| ABXDPP | CHAR | $3 | “Sim” (Yes), “Nao” (No) | Antibiotics given as results of positive DPP result? |
| JATOMABX | CHAR | $3 | “Sim” (Yes), “Nao” (No) | Was patient already taking antibiotics prior to DPP assay? |

#### Table *DPP_RefClinicalDiagnostics (N=57)*

#### *Reference clinical diagnostic laboratory results*

#### Note: Labs obtained solely at the discretion of the treating physician

| **VARIABLE** | **Field Type** | Format/Value | **Special Info** | **PROMPT** |
| --- | --- | --- | --- | --- |
| ID | NUMBER | #### |  | ID number |
| SOROLEPTO | CHAR | $15 | “Reagente” (Reactive),  “Nao reagente” (Non-reactive), “Indeterminate” (Indeterminate) | Serology for leptospirosis |
| SORODENGIGM | CHAR | $15 | “Reagente” (Reactive),  “Nao reagente” (Non-reactive), “Inconclusivo” (Inconclusive) | Dengue IgM |
| DENGISOLAM | CHAR | $15 | “Positive” (Positive), “Negativo” (Negative) | Dengue viral isolation |
| DENGSOROTIPO | CHAR | $6 | “DenV-1”,  “DenV-2”,  “DenV-3” | Dengue serotype |
| NS1DENG | CHAR | $15 | “Reagente” (Reactive),  “Nao reagente” (Non-reactive) | NS1 dengue antigen |
| HAVTOT | CHAR | $15 | “Reagente” (Reactive),  “Nao reagente” (Non-reactive) | Anti HAV total |
| HAVIGM | CHAR | $15 | “Reagente” (Reactive),  “Nao reagente” (Non-reactive), “Mat. Insuficiente” (Insufficient material) | Anti HAV IgM |
| AGHBS | CHAR | $15 | “Reagente” (Reactive),  “Nao reagente” (Non-reactive), “Inconclusivo” (Inconclusive) | AgHBS |
| HBCTOT | CHAR | $15 | “Reagente” (Reactive),  “Nao reagente” (Non-reactive) | Anti HBC total |
| ANTIHCV | CHAR | $15 | “Reagente” (Reactive),  “Nao reagente” (Non-reactive) | Anti HCV |
| SOROHIV | CHAR | $15 | “Reagente” (Reactive),  “Nao reagente” (Non-reactive) | HIV 1/2 serology |
| CMVIGG | CHAR | $15 | “Reagente” (Reactive),  “Nao reagente” (Non-reactive), “Inconclusivo” (Inconclusive), “Indeterminado” (Indeterminate) | Anti CMV IgG |
| CMVIGM | CHAR | $15 | “Reagente” (Reactive),  “Nao reagente” (Non-reactive) | Anti CMV IgM |
| EBVIGG | CHAR | $15 | “Reagente” (Reactive),  “Nao reagente” (Non-reactive) | Epstein-Barr IgG |
| EBVIGM | CHAR | $15 | “Reagente” (Reactive),  “Nao reagente” (Non-reactive) | Epstein-Barr IgM |
| HERPIGG | CHAR | $15 | “Positivo” (Positive), “Negativo” (Negative) | Anti herpes 1/2 IgG |
| HERPIGM | CHAR | $15 | “Positivo” (Positive), “Negativo” (Negative) | Anti herpes 1/2 IgM |
| HEMOCX | CHAR | $15 | “Positivo” (Positive), “Negativo” (Negative) | Blood culture |
| HEMOCXBACT | CHAR | $255 | If HEMOCX=”Positivo” (Positive) | Bacterial isolate |
| HEMOCXOBS | CHAR | $255 |  | Blood culture notes |
| CHAGASEIE | CHAR | $15 | “Reagente” (Reactive),  “Nao reagente” (Non-reactive) | Chagas EIE |
| CHAGASHA | CHAR | $15 | “Reagente” (Reactive),  “Nao reagente” (Non-reactive) | Chagas HA |
| DIAGSUP1 | CHAR | $255 | “Leptospirose” (Leptospirosis), “Dengue” (Dengue), “Hepatite A” (Hepatitis A), “Febre tifoide” (Typhoid), “EBV” (Mono), “Nenhum” (None) | Diagnosis #1 supported by reference lab results |
| DIAGSUP2 | CHAR | $255 | “Leptospirose” (Leptospirosis), “Dengue” (Dengue), “Hepatite A” (Hepatitis A), “Febre tifoide” (Typhoid), “EBV” (Mononucleosis), “Nenhum” (None) | Diagnosis #2 supported by reference lab results |

#### Table *DPP_Result_FSB (N=97)*

#### *Visual DPP results for finger stick blood (FSB)*

#### Note: One enrollee record missing due to precipitous death upon enrollment

| **VARIABLE** | **Field Type** | Format/Value | **Special Info** | **PROMPT** |
| --- | --- | --- | --- | --- |
| ID | NUMBER | #### |  | ID number |
| PDLEITOR1 | CHAR | $2 | “N1”, “N2”, “P1”, “P2”, “P3”  (N1 or N2 = Negative; P1, P2, or P3 = Positive) | Visual DPP result for operator #1 for FSB |
| PDLEITOR2 | CHAR | $2 | “N1”, “N2”, “P1”, “P2”, “P3”, “missing”  (N1 or N2 = Negative; P1, P2, or P3 = Positive) | Visual DPP result for operator #2 for FSB |
| PDDISC | CHAR | $3 | “Sim” (Yes), “Nao” (No) | Visual DPP result discordant between 2 operators for FSB |
| PDLEITOR3 | CHAR | $2 | “N1”, “N2”, “P1”, “P2”, “P3”, “missing”  (N1 or N2 = Negative; P1, P2, or P3 = Positive) | Visual DPP result for operator #3 for FSB |
| PDRESFINAL | CHAR | $3 | “POS”, “NEG” | Final visual DPP result for FSB (agreement between at least 2 of 3 operators) |

#### Table *DPP_Result_VWB (N=98)*

#### *Visual DPP results for venous whole blood (VWB)*

| **VARIABLE** | **Field Type** | Format/Value | **Special Info** | **PROMPT** |
| --- | --- | --- | --- | --- |
| ID | NUMBER | #### |  | ID number |
| STVLEITOR1 | CHAR | $2 | “N1”, “N2”, “P1”, “P2”, “P3”  (N1 or N2 = Negative; P1, P2, or P3 = Positive) | Visual DPP result for operator #1 for VWB |
| STVLEITOR2 | CHAR | $2 | “N1”, “N2”, “P1”, “P2”, “P3”, “missing”  (N1 or N2 = Negative; P1, P2, or P3 = Positive) | Visual DPP result for operator #2 for VWB |
| STVDISC | CHAR | $3 | “Sim” (Yes), “Nao” (No) | Visual DPP result discordant between 2 operators for VWB |
| STVLEITOR3 | CHAR | $2 | “N1”, “N2”, “P1”, “P2”, “P3”, “missing”  (N1 or N2 = Negative; P1, P2, or P3 = Positive) | Visual DPP result for operator #3 for VWB |
| STVRESFINAL | CHAR | $3 | “POS”, “NEG” | Final visual DPP result for VWB (agreement between at least 2 of 3 operators) |
| PD1STV1RESFINAL | CHAR | $3 | (Either VWB or FSB was Positive = POS) | Final visual DPP result for FSB and VWB combined (agreement between at least 2 of 3 operators) |

#### Table *DPP_Clinical (N=97)*

#### *Clinical information for enrolled patients*

#### Note: One enrollee record missing due to precipitous death upon enrollment

| **VARIABLE** | **Field Type** | Format/Value | **Special Info** | **PROMPT** |
| --- | --- | --- | --- | --- |
| ID | NUMBER | #### |  | ID number |
| AGE | NUMBER | ## |  | Age of patient |
| SEX | CHAR | $6 | “Male”, “Female” | Sex of patient |
| DAYSSYMPTOMS | NUMBER | ## |  | Days of symptoms prior to enrollment |
| ANTIBIOTICSPRIOR | CHAR | $7 | “Yes”,”No”,”Unknown” | Did patient take antibiotics prior to enrollment? |
| JAUNDICE | CHAR | $3 | “Yes”,”No” | Did patient have jaundice? |
| HEMOPTYSIS | CHAR | $3 | “Yes”,”No” | Did patient have hemoptysis? |
| ARI+JAUNDICE |  |  | “Yes”,”No” | Did patient have both jaundice and acute renal insufficiency? |
| ICU | CHAR | $3 | “Yes”, “No” | Was patient admitted to the ICU? |
| MAXCREATININE | NUMBER | ##.# |  | Highest creatinine during hospitalization |
| MAXALT | NUMBER | #### |  | Highest ALT during hospitalization |
| MAXTBILI | NUMBER | ##.# |  | Highest total bilirubin during hospitalization |
| PLATELETS | NUMBER | ###### |  | Lowest platelet count during hospitalization |
| PAIREDSERA | CHAR | $3 | “Yes”, “No” | Were both acute- and convalescent-phase sera collected? |
| HEMOCULTURE | CHAR | $8 | “Positive”,”Negative” | Result of hemoculture |
| MATRESULT | CHAR | $13 | “Confirmed”, “Not confirmed”, “Probable” | Result of MAT |
| ELISARESULT | CHAR | $12 | “Reactive”, “Non reactive”, “Probable” | Result of IgM-ELISA |
| DISPOSITION | CHAR | $18 | “Discharged to home”, “Transferred”, “Died” | Disposition at time of discharge from hospital for enrolled patients |
